# Intubation of the Larynx

**Published:** 1886-08

**Authors:** F. E. Waxham

**Affiliations:** Professor of Diseases of Children, College of Physicians and Surgeons, Chicago; 3449 Indiana Avenue


### INTUBATION OF THE LARYNX AS A SUBSTITUTE FOR TRACHEOTOMY IN THE TREATMENT OF PSEUDO-MEMBRANOUS LARYNGITIS; WITH A REPORT OF EIGHTY-THREE CASES.

*By* F. E. WAXHAM, M. D., *Professor of Diseases of Children, College of Physicians and Surgeons, Chicago.*

[Read before the Chicago Medical Society.]

A little over one year ago it was my privilege to present to this SOCIETY Dr. O'Dwyer's instruments for intubation, and to illustrate the operation upon the cadaver. The instruments were then imperfect, and a few trials and accidents proved the necessity for various modifications. First, the tubes were plain, with no shoulders, and they were too frequently rejected while coughing or vomiting. There was nothing to keep them in position but their length and weight.

Again, the heads of the tubes were too small, and after losing one in the trachea we were thoroughly convinced of the necessity for a change. The first important modification consisted in the addition of a shoulder to make them self-retaining. This shoulder consisted of a slight swelling or enlargement at the centre of the tube. Still they were not self-retaining, being frequently rejected by strong and vigorous children. The diameter was still further increased, and although they will now occasionally be rejected, yet they are as large as is consistent with safety. Another modification consisted in the enlargement of the head of the tube to prevent its falling into the trachea. A third, and, I believe, a very important modification,* has been the construction of the tubes with thinner walls, so that they are lighter and the calibre larger, giving greater breathing space and a better opportunity for the expulsion of false membrane. These tubes, which I now have the honor of presenting to you, will illustrate the various changes that have been made. In addition to these tubes for children I wish to present an adult tube which Dr. O'Dwyer has very kindly sent me. You will notice the great size and weight of this tube.†

The first extractor, and the one presented to you a year ago, consisted of an instrument with two jaws, opened and closed by a spring, one of the jaws entering the tube and the other passing outside. By pushing on the spring the tube is grasped between them.

Dr. O'Dwyer has had an extractor constructed on a somewhat different principle. The jaws while closed are passed into the tube and by pressing on the spring they are separated, thus holding the tube while it is extracted. This extractor has been very kindly furnished me by Hugo Keller, of New York.

---

* Made by Sharp and Smith, of Chicago.

† Which would seem objectionable, but which Dr. O'Dwyer claims to be an advantage, as it prevents expulsion.

No change has been made in the introducing instrument or the method of placing the tube.

A few words, however, in regard to the manner of introducing the tube may not be out of place. The operation has been so frequently described that it is unnecessary to go into details. I wish simply to refer to the relation of the index finger and the end of the tube while it is being introduced; a practical point which will be of great service to the inexperienced. There are two methods of placing the tube: we may introduce the index finger of the left hand until we feel the aperture of the larynx and then guide the tube into it, or we may simply reach the epiglottis, passing the end of the tube gently over it and then making an abrupt turn, when the tube, if in the median line, will slip into the larynx. The practical point is this: when the index finger has been introduced, the end of the tube, in either case, should be passed under the finger, not over it or by the side of it, but directly under it. In extracting the tube, the finger should be introduced over the epiglottis until it reaches the top of the tube; we then feel for the opening of the tube, and the extractor is introduced under the finger in exactly the same manner.

I would here *insist* upon the importance of practice upon the cadaver before attempting the operation upon the living child. While the expert will occupy scarcely five seconds in placing the tube, and with very little irritation and no injury, yet the inexperienced will often completely fail, causing the little patient great distress, the friends agony, and the physician great embarrassment. Indeed repeated and unsuccessful attempts upon the frightened, struggling, suffocating child are nothing short of cruelty. On two occasions it has been my misfortune to witness unfavorable terminations of apparently hopeful cases from detatchment of large masses of membrane below the tube. As our failures are oftentimes more instructive to us than our successes a history of one of these cases may not be uninteresting.

On March 8th I was called to see a boy, eight years old, in the last agonies of suffocation from diphtheritic laryngitis. Intubation was quickly performed and with prompt and entire relief to the distressing symptoms.

| | | | | | |
|---|---|---|---|---|---|
| | 4:30 P. M., | pulse, 130— | temp., 101— | respiration, | 24. |
| | 9:00 " | " 112— | " 102— | " | 24. |
| March 9th, | 9:00 A. M., | " 120— | " 102— | " | 20. |
| | 5:00 P. M., | " 112— | " 103— | " | 24. |
| | 9:00 " | " 110— | " 101— | " | 24. |
| " 10th, | 9:00 A. M., | " 112— | " 101— | " | 24. |

The patient seemed bright and perfectly comfortable, and took an abundance of nourishment without difficulty. There was every indication of a favorable termination. At about 4 o'clock, March 10th, a little over two days after the operation was performed, he had a "choking spell," but before my arrival he had succeeded in expelling a piece of membrane and was perfectly easy. Careful auscultation, however, revealed a bronchial complication. A mustard poultice was applied to the chest, followed by flaxseed poultices, and an expectorant mixture given. Fearing another attack of suffocation from detachment of membrane below the tube, I hastened home to make arrangements for remaining with the child all night. Hardly had I reached home before I was again summoned in the greatest of haste, the messenger stating that the boy was "choking to death." Upon arriving a few moments later I found the message too true, and upon entering the room the father exclaimed, "Oh, Doctor! you are too late, he is dead." Respiration had ceased and he was blue and pulseless. Nevertheless artificial respiration was resorted to and in a few moments he was resuscitated, although the respiration was labored and imperfect. The tube was at once extracted; during the operation the child passing into a convulsion. Chloroform was given to control the convulsion and help was summoned. In response to my urgent request for aid Drs. McWilliams and Sherry kindly came to my assistance. The child was placed upon the table, the trachea opened and a mass of membrane several inches in length removed. The patient revived, took nourishment well and seemed comfortable. There was, however, a rapid extension of the membranous exudate and the child died within twenty-four hours from bronchial obstruction. Another case terminated fatally in much the same manner, from peeling up of membrane directly after the removal of the tube.

This indeed is a danger that may be justly dreaded. In order to overcome it I have devised a tracheal forceps with which we may enter the trachea, by the way of the mouth, and remove the mass of membrane causing the obstruction.

There is one other source of danger. Occasionally a child will utterly refuse to take nourishment, and rectal enemas fail to support the patient sufficiently. To obviate this danger, I have had constructed a feeding-flask, consisting of a nursing bottle with a tube extending from it to the bulb of a Davidson syringe; this in turn is connected with an œsophageal tube. The tube is introduced into the œsophagus, and by pressing upon the bulb the contents of the bottle may be quickly transferred to the stomach. †

In considering whether we have a substitute for tracheot-

---

* Made by Sharp & Smith. † Made by E. H. Sargent & Co.

omy in tubage of the larynx, the first and most important question that will arise will be in regard to the comparative success of the two methods. Our text-books generally give one out of three as the average proportion of recoveries from tracheotomy, but that this percentage is too high, few I am sure will deny. It must be remembered that these results are obtained from reports of a few of our most successful tracheotomists. The thousand physicians who have met with inferior success never publish their cases and are never heard from excepting when they raise their voices against early operations. I have, myself, met with discouraging results with tracheotomy, notwithstanding that every patient had the most watchful care and attention, I know of one physician, not of this city, however, who has performed tracheotomy fifty times with but two recoveries, and another, noted for his skill as a surgeon, who has operated twenty times without saving a single patient, and another who has operated fourteen times without success, and another eight times without a recovery, and still another fifteen times with but one recovery, making one hundred and seven cases with but three recoveries.

I know of one of our eminent surgeons who delegates the operation to others when called upon to perform it on account of his loss of confidence in the procedure. Knowing these facts too well, I have often wondered why our text-books should have placed so high a value upon tracheotomy, and indeed have sometimes questioned whether the operation is justifiable.

In order to obtain the true status of tracheotomy in Chicago I have by personal inquiry, and by postal card, collected the following statistics, and have been surprised at the result. These inquiries disclose the fact that while we have many physicians who rarely meet with success and who have no faith in the operation, yet there are others who have done remarkable work.

These statistics show that one physician has performed the operation

| | | | | |
|---|---|---|---|---|
| | 3 | times with | 3 | recoveries. |
| Another | 7 | times with | 3 | recoveries. |
| " | 33 | " " | 9 | " |
| " | 25 | " " | 5 | " |
| " | 30 | " " | 6 | " |
| " | 12 | " " | 5 | " |
| " | 20 | " " | 0 | " |
| " | 14 | " " | 0 | " |
| " | 8 | " " | 0 | " |
| " | 15 | " " | 1 | " |
| " | 16 | " " | 1 | " |
| " | 15 | " " | 3 | " |
| " | 9 | " " | 1 | " |
| " | 1 | " " | 0 | " |
| " | 1 | " " | 0 | " |
| " | 4 | " " | 0 | " |
| " | 1 | " " | 0 | " |
| " | 3 | " " | 0 | " |
| " | 8 | " " | 2 | " |
| " | 1 | " " | 1 | " |
| " | 2 | " " | 0 | " |
| " | 5 | " " | 1 | " |
| " | 3 | " " | 1 | " |
| " | 4 | " " | 1 | " |
| " | 1 | " " | 0 | " |
| " | 1 | " " | 0 | " |
| " | 3 | " " | 0 | " |
| " | 2 | " " | 1 | " |

| | | | | |
|---|---|---|---|---|
| | 3 | times with | 3 | recoveries. |
| Another | 2 | times with | 0 | recoveries. |
| " | 4 | " " | 2 | " |
| " | 1 | " " | 0 | " |
| " | 3 | " " | 1 | " |
| " | 2 | " " | 1 | " |
| " | 5 | " " | 1 | " |
| " | 3 | " " | 2 | " |
| " | 3 | " " | 2 | " |
| " | 1 | " " | 0 | " |
| " | 3 | " " | 1 | " |
| " | 1 | " " | 0 | " |
| " | 2 | " " | 0 | " |
| " | 4 | " " | 1 | " |
| " | 2 | " " | 0 | " |
| " | 2 | " " | 0 | " |
| " | 2 | " " | 1 | " |
| " | 6 | " " | 1 | " |
| " | 1 | " " | 1 | " |
| " | 3 | " " | 0 | " |
| " | 1 | " " | 0 | " |
| " | 1 | " " | 0 | " |
| " | 3 | " " | 0 | " |
| " | 2 | " " | 0 | " |
| " | 2 | " " | 0 | " |
| | 306 | | 58 | |

Making a total of 306 cases with 58 recoveries, or a percentage of 18.95.

In one hundred and thirty-eight cases the ages of the patients were as follows:

| | | | | | |
|---|---|---|---|---|---|
| 1 | was | 2 yrs. and 2 mo. | 9 | were | 2 yrs. |
| 1 | " | 7 mo. | 2 | " | 2 " and 4 mo. |
| 1 | " | 13 " | 4 | " | 2½ " |
| 1 | " | 12 " | 19 | " | 3 " |
| 1 | " | 20 " | 7 | " | 3½ " |
| 1 | " | 2 yrs. and 5 mo. | 14 | " | 4 " |
| 1 | " | 2 " " 3 " | 16 | " | 5 " |
| 1 | " | 3 " " 3 " | 3 | " | 5½ " |
| 1 | " | 4½ " | 16 | " | 6 " |
| 1 | " | 5 " and 7 mo. | 6 | " | 7 " |
| 1 | " | 6½ " | 8 | " | 8 " |
| 1 | " | 7½ " | 5 | " | 9 " |
| 1 | " | 19 " | 4 | " | 10 " |
| 1 | " | 24 " | 2 | " | 12 " |
| 6 | were | 18 mo. | 3 | " | 13 " |

This gives an average of 5 years and 1 month. Where the cause of death has been referred to, extension of the membrane into the bronchial tubes has been given most frequently.

In contrast to these statistics it is my pleasure to report 83 cases of intubation with 23 recoveries, or 27.71 per centum. Three of these cases were reported to me by Dr. C. P. Caldwell, five by Dr. Ingals, seven by Dr. Strong, ten by Dr. Richardson, and fifty-eight were under my own care. The ages were as follows:

| No. of cases. | Age. | Recoveries. | No. of cases. | Age. | Recoveries. |
|---|---|---|---|---|---|
| 1 | 9 mo. | 0 | 12 | 3 yrs. | 2 |
| 1 | 11 " | 0 | 1 | 3 " and 4 mo. | 1 |
| 1 | 13 " | 0 | 1 | 3 " " 6 " | 0 |
| 3 | 14 " | 1 | 11 | 4 " | 5 |
| 1 | 15 " | 0 | 1 | 4 " " 9 " | 1 |
| 1 | 16 " | 0 | 3 | 4½ " | 0 |
| 1 | 17 " | 0 | 9 | 5 " | 4 |
| 1 | 18 " | 1 | 6 | 7 " | 1 |
| 1 | 20 " | 1 | 1 | 7½ " | 0 |
| 7 | 2 yrs. | 2 | 2 | 8 " | 1 |
| 2 | 2 " and 1 mo. | 1 | 1 | 11 " | 0 |
| 2 | 2 " " 2 " | 1 | 7 | — | 0 |
| 2 | 2 " " 3 " | 0 | | | |
| 3 | 2 " " 6 " | 1 | 83 | | 23 |
| 1 | 2 " " 9 " | 0 | | | |

Average age, 3 years and 7 months.

It will be observed that 11 cases with 3 recoveries were under 2 years of age, 28 with 8 recoveries were under 3 years, 14 cases with 3 recoveries between 3 and 4 years, 15 cases with 6 recoveries between 4 and 5, 9 cases between 5 and 6 with 4 recoveries and 10 cases with 2 recoveries between 7 and 11 years.

It will also be observed that while the percentage of recoveries from tracheotomy in the 306 cases reported has been 18.95, that from intubation has been 27.71. This, too, represents, not simply recoveries from the operation but entire recoveries from the disease which rendered the operation necessary.

Of the 58 cases coming under my care, 20 were actually moribund when the operation was performed, many of them entirely unconscious, and 40 were bad cases of diphtheria, characterized by severe constitutional symptoms and extensive exudation in the pharynx as well as in the larynx. In 18 cases the exudation in the pharynx was slight, but in every case, without exception, false membrane was expelled, either in the form of muco-pus, shreds or casts. In every case the operation was performed to prevent impending suffocation and the cases pronounced hopeless without surgical interference. In addition to the recoveries cited above, the operation was perfectly successful in 18 others, although the patients died. Thus 4 died perfectly easy, before the removal of the tube, from the severity of the diphtheritic disease; 3 died easily from one to several days after the removal of the tube, from exhaustion incident to the disease; 1 died from paralysis of the heart; 1 from uræmic convulsions; 3 from pneumonia, resulting from hypostatic congestion, and 6 from pneumonia, resulting from unfavorable surroundings. These cases added to those where entire recovery occurred and the result 41, or 49.39 per centum represents the proportion of cases in which the operation was successful and entirely satisfactory; the remaining cases dying generally from extension of the membrane into the bronchi.

Many of these cases were delegated to me because they were considered too young or too unfavorable for tracheotomy. Many of them were young, nursing infants; and although the operation can rarely succeed, yet the fact that we have been able to save one child but fourteen months old and another of twenty months should be an encouragement always to perform the operation, when indicated, without regard to age. One of these cases was so truly remarkable that a brief history may prove instructive.

May 5th I was called to perform intubation upon a nursing infant of fourteen months, a patient of Dr. Lawless. The infant had just recovered from measles when taken with diphtheria. The pharyngeal deposit was slight, but quickly extended into the larynx. Prof. Steele, who assisted in the operation, expressed the opinion that the infant could live but a few hours without an operation. Intubation was quickly performed, and with instant relief to the urgent symptoms.

Considerable mucus and softened membrane were expelled, and the little patient at once passed into a quiet sleep. Milk was drawn from the breast and given the infant from a spoon for the first two days, after which time it was able to nurse. For two days the little patient did remarkably well, appearing bright and strong and coughing with considerable force.

With the assistance of Drs. Lawless and Clendenning the tube was removed after being in position for two days, but it was necessary to at once reintroduce it. On the third day the strength began to fail, and the patient seemed to be doing poorly. Mucus and softened membrane would collect in the tube and the infant had not sufficient strength to expel it. At the expiration of the third day the tube was removed, thoroughly cleansed and reintroduced. The infant was in a bad condition, and little hope of recovery was entertained. At about 2 A. M., May 9th, I was called to see the little patient again, and found that it had been having convulsions. The breathing was embarrassed and rapid, and evidently indicated obstruction of the tube. The tube was again removed, after having been in position three and a half days. The respiration, although very rapid, numbering 70 per minute, was comparatively easy, and the tube was not reintroduced. Warm flaxseed poultices were applied to the chest, and the next morning there was marked improvement. Convalescence proceeded slowly but surely, and in a few days the little patient was entirely out of danger and made a perfect recovery.

Many of these cases have been moribund, pulseless, limp, and unconscious when the operation was performed. The history of a single case will illustrate the unfavorable condition of many of these cases.

May 6th I was called by Dr. Clendenning to perform intubation upon a child three years old. Upon arriving we found the child dying. It was blue, cold, pulseless and unconscious. An occasional gasp was the only sign of life. A tube was quickly introduced, not even producing gagging or coughing. Artificial respiration was performed, the child thoroughly rubbed, and after working vigorously for half an hour we were gratified to see the child open its eyes and look about. A few teaspoonfuls of brandy now excited coughing and we soon had the throat free from mucus and softened membrane. The child took nourishment well, the pulse returned, the warmth returned to the surface and the little patient was left sleeping quietly and comfortably. The next day the child was bright and happy, breathing easily, and no one would have imagined that there was a tube two inches in length in its throat, or that the day before it was at death's door. The patient did remarkably well for three days, giving every promise of recovery. The membrane, however, gradually extended into the bronchi, and the child died four days after the operation was performed. Although the prognosis is far more unfavorable where the patient is moribund, yet that they may be perfectly resuscitated I have proved over and over again, and it has been my pleasure to save one patient in such a desperate condition when the operation was performed. In conclusion I would say, that intubation possesses many advantages over tracheotomy. The ease and rapidity with which the operation can be performed, the perfect comfort of the patient and the rapid convalescence after the removal of the tube are in great contrast to tracheotomy. In addition to this, intubation is more successful. While the record of recoveries after tracheotomy in Chicago has been 18.95 per cent, that of intubation has been 27.71. Again, one of our most successful tracheotomists in Chicago has performed tracheotomy 33 times with 9 recoveries in a period of twenty years. That I have performed intubation more times and saved more lives in a single season is still further evidence of the superiority of the new operation over tracheotomy.

In closing this paper I would take occasion to thank the members of the profession of this city for the many courtesies extended to me. To the following gentlemen I am particularly indebted and would refer to them as to the accuracy of this report: Dr. Steele, Dr. Sullivan, Dr. Hatfield, Dr. Casselberry, Dr. Starkey, Dr. Behrend, Dr. H. T. Byford,

Dr. Richardson, Dr. Dahlberg, Dr. Appleby, Dr. Helm, Dr. Bosworth, Dr. Nelson, Dr. Kosakowski, Dr. Valin, Dr. Chas. E. Caldwell, Dr. Ogden, Dr. Holroyd, Dr. Pierpoint, of Englewood, Dr. Quine, Dr. Miller, of Kensington, Dr. Willard, Dr. C. P. Caldwell, Dr. Dyas, Dr. Berry, Dr. McGinnis, of Brighton, Dr. Hoadley, Dr. Nutt, of Marengo, Dr. Rea, Dr. Hempstead, Dr. Clark, of Rockford, Dr. Hall, Dr. Van Doozer, Dr. McWilliams, Dr. Parsons, Dr. Fenger, Dr. Storck, Dr. Lawless, Dr. Tillosten, Dr. Clendening, Dr. Collins, Dr. Crane, Dr. H. A. Johnson, Dr. Manheimer, Dr. Webb, Dr. Able, Dr. Simons, Dr. McDonald, Dr. Hollister, Dr. Goodall, Dr. R. G. Bogue, Dr. Shepherd, Dr. E. F. Ingals, Dr. A. B. Strong.

3449 Indiana Avenue.

